# Recurrent multiple eye muscle palsy as a first sign of sarcoidosis

**DOI:** 10.3205/oc000229

**Published:** 2023-11-07

**Authors:** Isabel Deboutte, Daisy Godts, Michel Van Lint

**Affiliations:** 1 Department of Ophthalmology, Antwerp University Hospital, Edegem, Belgium; 2 Medical Science Department, University of Antwerp, Belgium

**Keywords:** sarcoidosis, neurosarcoidosis, eye muscle palsy

## Abstract

**Purpose:**

To report a case of (neuro)sarcoidosis presenting solely with recurrent cranial nerve palsies in a 57-year-old Caucasian female

**Methods:**

Case report with clinical imaging

**Results:**

A 57-year-old female first presented with a right sixth nerve palsy, which resolved spontaneously after 6 months. Three years later she was diagnosed with a sixth nerve palsy in the fellow eye followed by a complete palsy of the left third cranial nerve four months after. Medical history consisted of migraine and hypercholesterolemia. Further neurological and ophthalmic work-up was unrevealing at first. After repeated magnetic resonance imaging, an enhancing lesion in the left cavernous sinus was seen, which was initially diagnosed as a meningioma. However, imaging of the chest revealed an image of sarcoidosis, and the lesion and ophthalmoplegia of the left eye disappeared with systemic corticosteroid treatment.

**Discussion:**

Sarcoidosis is the ultimate imitator and the possibility of neurosarcoidosis must be taken into account when presented with unexplained ophthalmoplegia. Neurosarcoidosis has imaging properties very similar to other diseases such as a meningioma, and misdiagnosis occurs easily. Spontaneous recovery of ophthalmoplegia can rarely occur in neurosarcoidosis.

## Introduction

Neurosarcoidosis is seen in up to 10% of sarcoidosis cases, with neuro-ophthalmic sarcoidosis being found in about a third of neurosarcoidosis cases [1]. Optic nerve and chiasmal involvement is the most common manifestation in neurosarcoidosis, but other cranial nerves, mostly the seventh, can be involved, as can almost all other parts of the nervous system [1], [2]. Involvement of other cranial nerves as the sole manifestation of sarcoidosis remains relatively rare [3]. We present a case of neuro-ophthalmic sarcoidosis appearing as multiple cranial nerve palsies over three years.

Recurrent cranial nerve palsies and eventually chest imaging showing enlarged hilar lymph nodes led to a possible diagnosis of neurosarcoidosis.

Diagnosis of neurosarcoidosis is difficult because of its broad range of clinical manifestations, the close resemblance to other neuro-inflammatory diseases and because definitive diagnosis warrants pathological confirmation of granulomatous disease in biopsy of neurological tissue [4]. This makes of sarcoidosis the ultimate imitator. Therefore, unusual cases of sarcoidosis serve to remind us of this diagnosis. Moreover, the radiological resemblance of neurosarcoidosis to other pathologies must be emphasized to avoid unnecessary neurosurgery and distress for the patient.

## Case description

**Figure 1:**
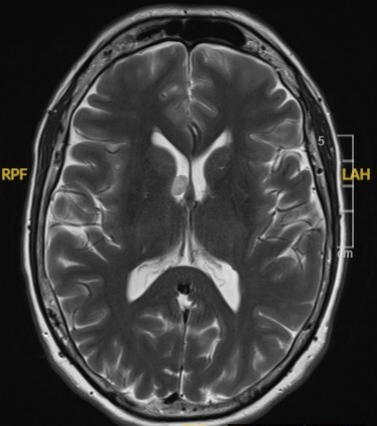
MRI scan (2014) showing lesion right lateral ventricle

A 57-year-old Caucasian woman presented to our department in April 2014 with sudden binocular horizontal diplopia. There were no other neurological symptoms, no recent infection or trauma. Apart from a refractive procedure (LASIK) years before, she had no ophthalmological history. Medical history consisted of migraine and hypercholesterolemia for which she was taking Atorvastatin 10 mg. No other cardiovascular risk factors could be withheld. She fulfilled an administrative occupation at the time. On ocular examination there was an esotropia (ET) of 10° of the right eye (RE) and an abnormal head turn to the right side. Eye motility exam showed a –4 abduction limitation of the RE due to a complete right sixth nerve palsy. There was no nystagmus. Alternate cover testing (ACT) showed 45 Δ ET with right fixation (RF) and 15 Δ ET with left fixation (LF) at 30 cm, and 45 Δ ET and 18 Δ ET respectively at 6 m. Best corrected visual acuity (BCVA) as measured with EDTRS chart was 1.0 in both eyes (BE). Intra-ocular pressure (IOP) was normal. Pupils were equal and reactive to light. Eyelids were symmetrical; there was no eyelid retraction or ptosis. Clinical corneal reflex testing revealed a hypoesthetic cornea on the left side. Apart from this, anterior and posterior segment findings were normal. Blood pressure was normal. Laboratory analysis revealed no abnormalities: blood cell count was normal, liver and renal function test were normal, serology (including syphilis and *Borrelia burgdorferi*) was negative, as was antibody testing (ANCA, ANA, anti-phospholipids, IgG). The patient was sent for further neurological investigation, but aside from the sixth nerve palsy the neurological exam including lumbar puncture appeared normal. Magnetic resonance imaging (MRI) without gadolinium contrast of the brain and orbit showed a small incidental lesion (7x10 mm) (Figure 1 (Fig. 1)) in the right lateral ventricle (DD astrocytoma, hamartoma, subependymoma), not responsible for the right sixth nerve palsy. This lesion remained unchanged during follow-up with gadolinium-enhanced MRI scans 6 and 12 months later.

Tentative diagnosis of a microvascular nerve palsy was made. The patient was treated with abduction exercises, occlusion of the left eye (LE) and botulinum toxin type A injection (BTXA) 2.5 units (U) in the right medial rectus in May 2014. She recovered full motility over the course of 6 months but a small ET at distance remained (10 Δ ET RF=LF), for which prism glasses were prescribed (5 Δ base out for both eyes). 

In January 2017 she presented with a second episode of sudden diplopia caused by a sixth nerve palsy, but this time in the left eye. She complained of a headache and had a cold. She now presented with an ET and abduction deficit of the left eye. ACT revealed 20 Δ ET with RF, 50 Δ ET with LF at 30 cm, and 35 Δ ET and >50 Δ ET respectively at 6 m. Abduction limitation worsened to –4 over 4 weeks. Apart from this, the ophthalmological, general and neurological exam was normal. 

Another MRI without gadolinium contrast did not reveal new findings. The lesion in the right lateral ventricle appeared unchanged. Treatment was initiated with Fresnel prism, duction exercised and BTXA 5U in left medial rectus in February 2017. The neurologist started Asaflow 80 mg (acetylsalicylic acid). The patient saw some improvement at first when in May 2017 she presented with ptosis occurring over 1 week and a pressure feeling in the left side of the face. At first the hypothesis was made that this was a side effect of the BTXA injection. However, over the following 4 weeks the ptosis worsened and she developed an exotropia (XT) and a mydriatic fixed pupil on the left eye, accounting for a complete palsy of the left third cranial nerve. ACT showed 20 Δ XT with RF and 70 Δ XT with LF at near and 25 Δ XT and >70 Δ XT respectively at distance, as well as 6 Δ hypertropia (HT) of the right eye at distance. Motility exam showed –4 adduction, –4 depression and –4 elevation limitation in the left eye. A reduced corneal reflex and hypoesthesia in the V1 dermatome on the left side was noted. Sensibility in other dermatomes was normal.

**Figure 2:**
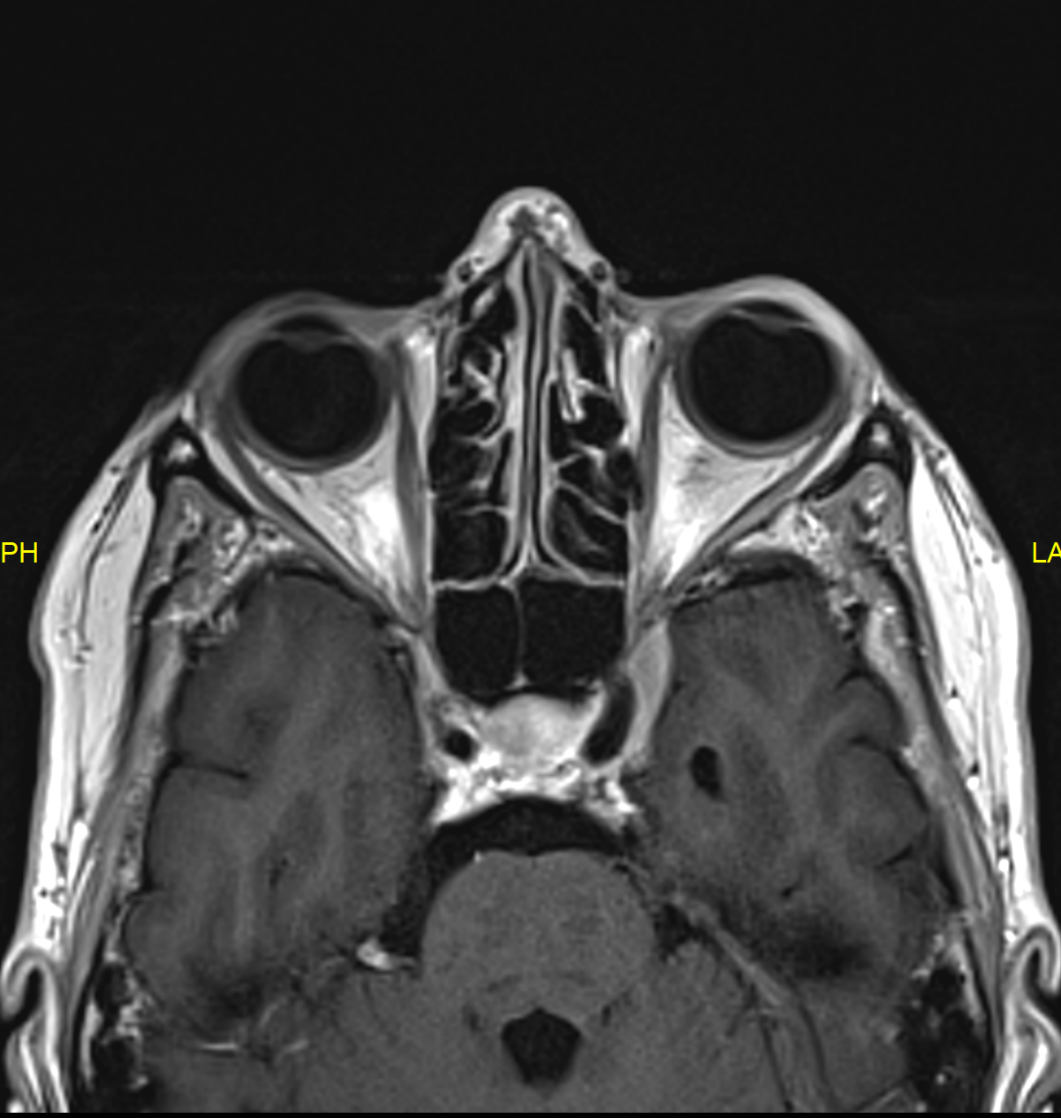
MRI scan (2017) showing dural enhancement at the left cavernous sinus

**Figure 3:**
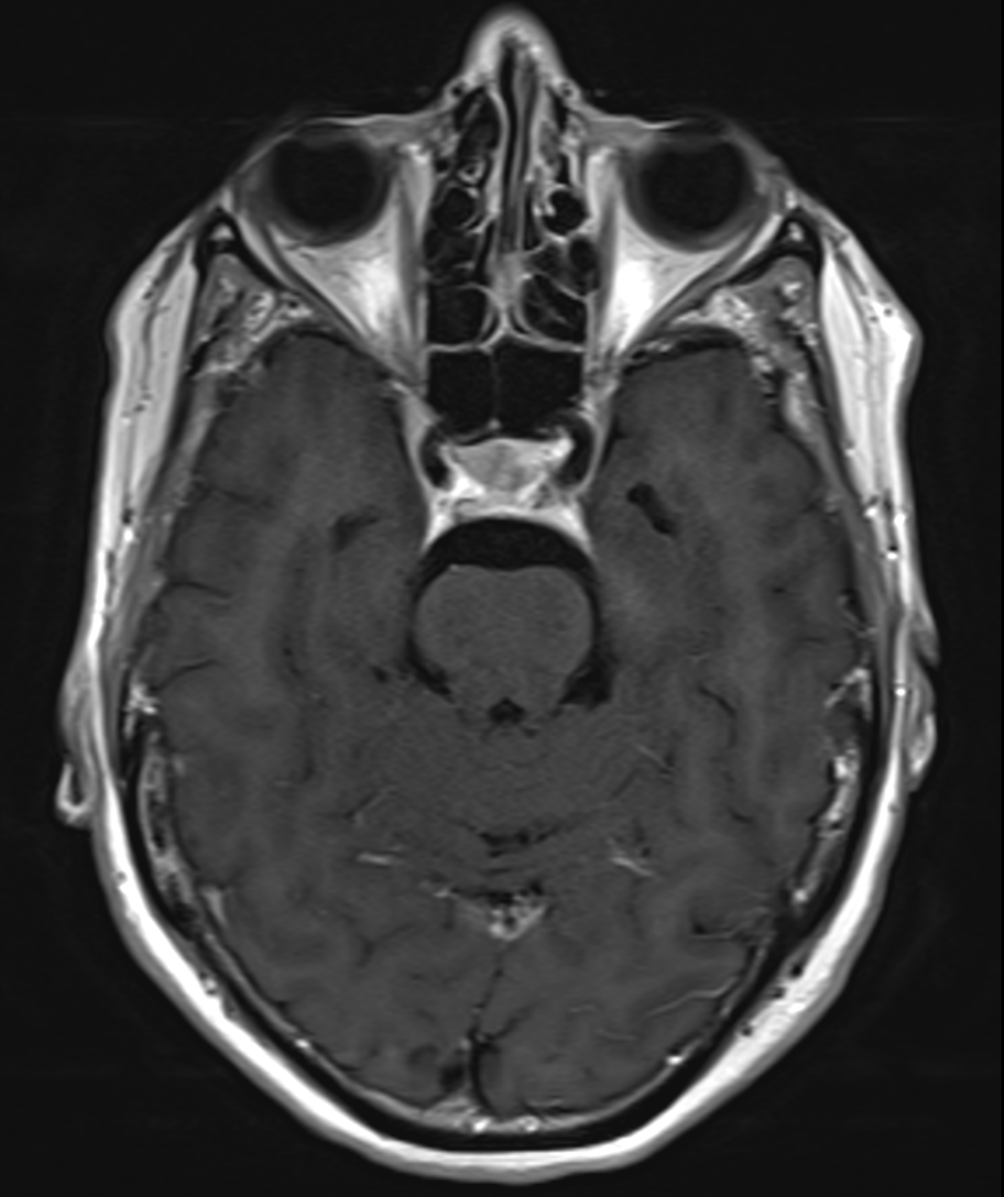
MRI scan (2014) at the level of the cavernous sinus

An urgent MRI with gadolinium contrast was performed and described a dural enhancement (5 mm thickness, antero-posterior (AP) over 2 cm) and dural tail sign at the left cavernous sinus in contact with the left internal carotid artery as with the left optic canal (Figure 2 (Fig. 2)). This new lesion was radiologically diagnosed as a meningioma and found to be explanatory for the third and fifth (V1) nerve palsy but was unable to explain the former cranial nerve palsies and had not been present at previous MRI scans, with and without gadolinium contrast (Figure 3 (Fig. 3)). 

**Figure 4:**
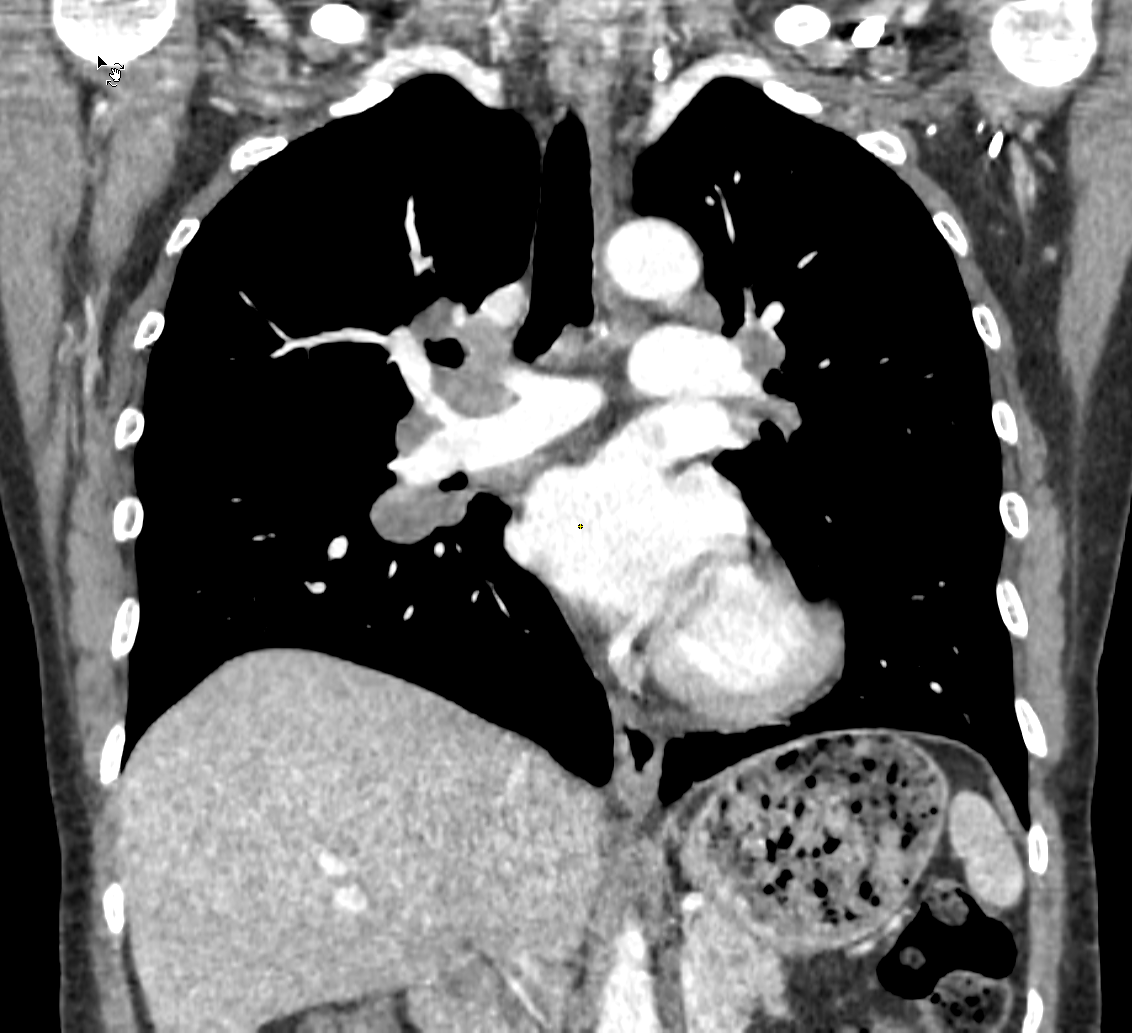
CT (2017) of the chest and upper abdomen showing mediastinal enlarged lymph nodes

Computed tomography (CT) of the thorax was performed in June 2017 to rule out other possible etiologies and showed enlarged mediastinal and hilar lymph nodes and various nodules in the lung parenchyma as well as in the upper abdomen. This image was suspicious of sarcoidosis (Figure 4 (Fig. 4)). Treatment with corticosteroids was started at 64 mg/day orally and then tapered to 4 mg/day over 2 weeks. 

Other organ involvement (cardial, pneumological) was not clinically present. To confirm the diagnosis of sarcoidosis, biopsy of the mediastinal or hilar lymph nodes was suggested, but the patient refused this. The diplopia and ptosis resolved over the next 2 months and the patient remained comfortable with her prism glasses. There was a complete resolution of the eye motility deficit. ACT showed 4 Δ exoforia (X) at near (RF=LF) and 4 Δ esoforia (E) (RF=LF) at distance.

**Figure 5:**
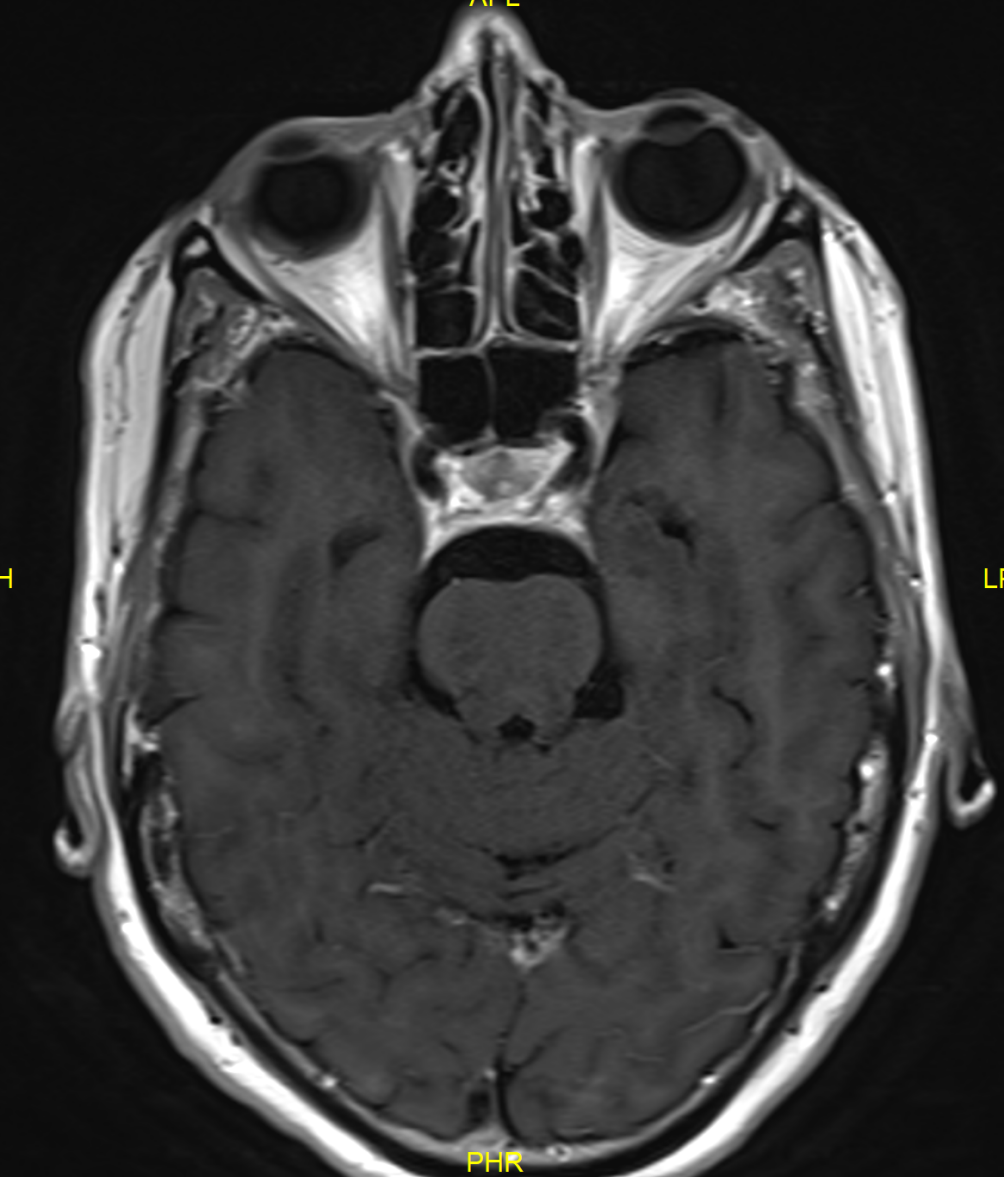
MRI (2019) at the level of the cavernous sinus

**Figure 6:**
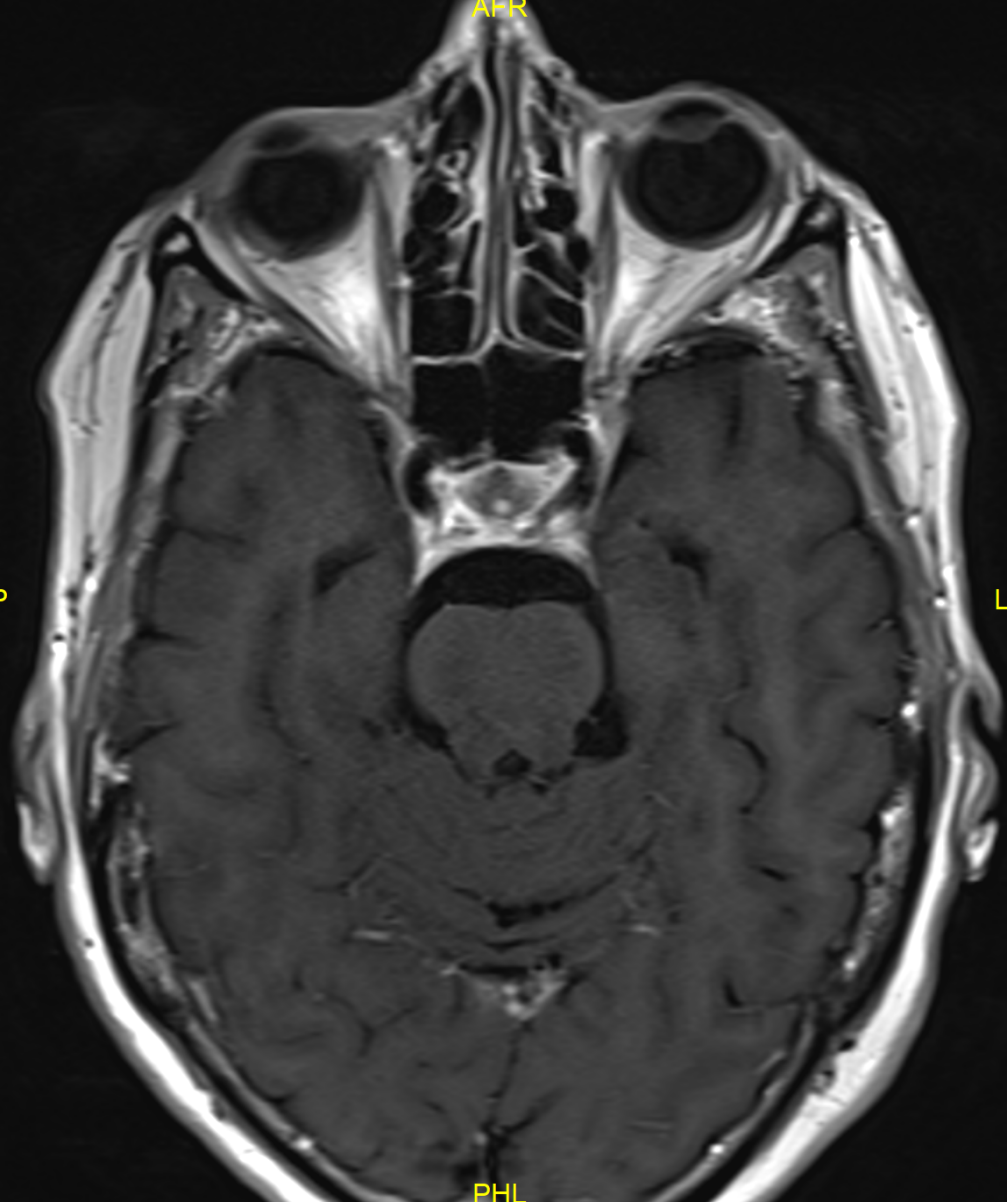
MRI (2020) at the level of the cavernous sinus

In January 2018 the patient complained of a fourth episode of diplopia due to discrete left sixth nerve palsy. Corticosteroid treatment was restarted at 64 mg and tapered slowly over the next 6 weeks. Gadolinium-enhanced MRI of the brain was conducted at six months interval and in May 2019 a decrease in the volume of the dural enhancement in the left cavernous sinus was described (Figure 5 (Fig. 5)). The last MRI in October 2020 showed almost no visible enhancement of the dura in the left cavernous sinus, confirming it not to be a meningioma, but an inflammation compatible with dural sarcoidosis (Figure 6 (Fig. 6)).

## Discussion

Sarcoidosis is an idiopathic inflammatory disease, infamous because of its various disease-mimicking abilities and the wide range of clinical manifestations in multiple systems and organs. The diagnosis of neurosarcoidosis was made only three years after the first onset of symptoms. Earlier diagnosis might have spared the patient investigations and anxiety and would have improved her quality of life during this time. 

The initial finding in this case was a complete right sixth nerve palsy in a Caucasian woman above the age of 50. Without further symptoms, signs or clues on additional testing, the diagnosis of sarcoidosis was extremely challenging at this point. Because of spontaneous recovery, a microvascular cause was suspected. The second episode of diplopia due to another sixth cranial nerve palsy was therefore equally falsely attributed to a microvascular cause. A third episode of diplopia with findings of a third cranial nerve palsy ultimately led to the diagnosis of neurosarcoidosis. A third nerve palsy secondary to microvascular disease has been reported but remains uncommon [5].

In patients presenting with recurrent cranial nerve deficits, the main differential diagnosis includes multiple sclerosis, neuroborreliosis, other central nervous system (CNS) inflammation or infection, increased intracranial pressure, lymphoma and neurosarcoidosis. Diagnostic testing includes serum and cerebrospinal fluid (CSF) analysis. Angiotensin converting enzyme (ACE) might be elevated but should not be regarded as a diagnostic test in patients with isolated neurosarcoidosis as it is nonspecific and not too sensitive [6]. It was not elevated in the discussed case. MRI is the gold standard for neurosarcoidosis imaging but may be negative, especially in non-enhanced imaging studies [2]. A wide range of findings has been described in neurosarcoidosis [7]. In our case, gadolinium-enhanced MRI showed a dural enhancement in the left cavernous sinus at the time of the third cranial nerve palsy. This lesion was radiologically diagnosed as a meningioma due to the characteristic imaging properties and a ‘dural tail’ sign (DTS). Initially regarded as pathognomic for a meningioma, it has become increasingly noted that the DTS can be present in many other conditions like dural sarcoidosis [8]. In the brain, leptomeningeal and dural sarcoidosis is seen in approximately 40% of patients with neurosarcoidosis [9], [10]. The cranial nerves may become involved via perineural spread from adjacent sites. The close proximity of the meninges in the cavernous sinus explains why the involvement of the cranial nerves is often in this location. Dural sarcoidosis and leptomeningeal involvement, although not typically combined, present with similar symptoms relating to cranial nerve involvement if present and more general symptoms like fatigue and headache [11]. The patient in this case complained of a headache, but this is a nonspecific symptom [2]. Imaging in dural sarcoidosis characteristically shows isointense diffuse dural thickening or focal dural masses on unenhanced T1-weighted images and hypointensity on T2-weighted images. Enhancement can be seen on gadolinium contrast-enhanced T1-MRI [7], [9]. This enhancement on T1-weighted image was seen in the discussed case. Unfortunately, these imaging characteristics are nonspecific and insufficient to differentiate neurosarcoidosis from other dural diseases including meningioma [9], [11]. There is a number of published cases of neurosarcoidosis mimicking meningioma on imaging studies, as occurred in the discussed case [11]. Accurate diagnosis is of great importance, since neurosurgical procedures can be avoided. 

Suspicion of sarcoidosis with confirmation of enlarged hilar lymph nodes on chest imaging led to a tentative diagnosis in this case. When diagnosis of neurosarcoidosis is suspected, extraneurologic evaluation is warranted [2]. Our patient did not display an extraneurological disease. Definitive diagnosis consists of pathological confirmation of granulomatous disease in biopsy of neurological tissue. Otherwise, a probable diagnosis can be made if biopsy was obtained from extraneurological tissue, as is most often the case with a biopsy of any accessible enlarged lymph nodes [4]. In this case, the patient refused lymph node biopsy.

Corticosteroids and other immune-suppressants like anti-TNF are considered the main therapy [12]. In general, oral corticosteroids are used for mild to moderate cases, while high-dose intravenous methylprednisolone is used in severe or refractory cases that fail to respond to oral corticosteroids [2]. Spontaneous remissions have been rarely reported in sarcoid optic neuropathy, facial nerve and vestibulocochlear nerve palsy [13]. A spontaneous recovery of a sixth nerve palsy, as occurred initially in this case, has not been described before in neurosarcoidosis [13]. 

With this case, we want to emphasize the importance of maintaining a high index of suspicion for neurosarcoidosis despite a negative workup. Although the initial MRI of the brain did not describe any suspicious lesions in this case, later MRI revealed findings of neurosarcoidosis, but diagnosis remained challenging. It is important to know that neurosarcoidosis has imaging properties very similar to other diseases such as a meningioma and that misdiagnosis occurs easily. Therefore, collaboration between ophthalmologists and neuroradiologists is important to assure accurate diagnosis. 

## Conclusion

The broad range of possible manifestations makes sarcoidosis the ultimate imitator. A high dose of suspicion is warranted in cases of recurrent cranial nerve palsy, despite an initial negative workup. Repeated gadolinium-enhanced MRI might be necessary.

Imaging features of neurosarcoidosis are nonspecific and include thickening and enhancement of the dura, closely resembling a meningioma. Although rare, spontaneous recovery of cranial nerve palsy can occur.

## Notes

### Competing interests

The authors declare that they have no competing interests.
